# Changes in motor unit behaviour across repeated bouts of eccentric exercise

**DOI:** 10.1113/EP092070

**Published:** 2024-09-03

**Authors:** Oliver Hayman, Paul Ansdell, Luca Angius, Kevin Thomas, Lauren Horsbrough, Glyn Howatson, Dawson J. Kidgell, Jakob Škarabot, Stuart Goodall

**Affiliations:** ^1^ Department of Sport, Exercise, & Rehabilitation, Faculty of Health and Life Sciences Northumbria University Newcastle upon Tyne UK; ^2^ School of Cardiovascular and Metabolic Health, BHF Glasgow Cardiovascular Research Center, College of Medical, Veterinary, and Life Sciences University of Glasgow Glasgow UK; ^3^ Water Research Group North West University Potchefstroom South Africa; ^4^ Monash Exercise Neuroplasticity Research Unit, Department of Physiotherapy, School of Primary and Allied Health Care, Faculty of Medicine, Nursing and Health Science Monash University Melbourne Australia; ^5^ School of Sport, Exercise and Health Sciences Loughborough University Loughborough UK; ^6^ Physical Activity, Sport and Recreation Research Focus Area, Faculty of Health Sciences North‐West University Potchefstroom South Africa

**Keywords:** fatigue, high‐density electromyography, motoneuron, muscle damage, recovery

## Abstract

Unaccustomed eccentric exercise (EE) is protective against muscle damage following a subsequent bout of similar exercise. One hypothesis suggests the existence of an alteration in motor unit (MU) behaviour during the second bout, which might contribute to the adaptive response. Accordingly, the present study investigated MU changes during repeated bouts of EE. During two bouts of exercise where maximal lengthening dorsiflexion (10 repetitions × 10 sets) was performed 3 weeks apart, maximal voluntary isometric torque (MVIC) and MU behaviour (quantified using high‐density electromyography; HDsEMG) were measured at baseline, during (after set 5), and post‐EE. The HDsEMG signals were decomposed into individual MU discharge timings, and a subset were tracked across each time point. MVIC was reduced similarly in both bouts post‐EE (Δ27 vs. 23%, *P *= 0.144), with a comparable amount of total work performed (∼1,300 J; *P *= 0.905). In total, 1,754 MUs were identified and the decline in MVIC was accompanied by a stepwise increase in discharge rate (∼13%; *P *< 0.001). A decrease in relative recruitment was found immediately after EE in Bout 1 versus baseline (∼16%; *P* < 0.01), along with reductions in derecruitment thresholds immediately after EE in Bout 2. The coefficient of variation of inter‐spike intervals was lower in Bout 2 (∼15%; *P *< 0.001). Our data provide new information regarding a change in MU behaviour during the performance of a repeated bout of EE. Importantly, such changes in MU behaviour might contribute, at least in part, to the repeated bout phenomenon.

## Abstract (highlights)

- **What is the central question of this study?**
  Is motor unit (MU) behaviour altered *during* the performance of an initial and repeated bout of eccentric exercise (EE)?
- **What is the main finding and its importance?**
  During the repeated bout of exercise, we observed a greater contribution from lower threshold MUs to force generation along with reduced firing rate variability. Results suggest an adaptation occurs *during* the performance of an initial bout of exercise and contributes to the accelerated recovery that occurs following the performance of a repeated bout of EE. Importantly, our data suggest that changes in MU behaviour contribute, partly, to the repeated bout phenomenon immediately upon performing a repeated bout of eccentric activity.

## 1 INTRODUCTION

Unaccustomed eccentric exercise (EE) causes symptoms of exercise‐induced muscle damage (EIMD; Peake et al., 2017), identified with symptomatic (muscle soreness, reduced muscular strength, and range of motion), systemic (leakage of intramuscular enzymes) and histological (myofibrillar disruptions) markers (Burt et al., 2020; Doma et al., 2017). Some of these responses become evident immediately following EE, often peaking between 24 and 96 h postexercise (Chen et al., 2021). Whilst most previous research has investigated the damage response on skeletal muscle (Doma et al., 2017), some work has shown central nervous system‐related changes (Minett & Duffield, 2014; Škarabot et al., 2019), along with the ability to activate muscle (i.e., voluntary activation; Goodall et al., 2017). When a repeated bout of EE is performed, the associated symptomatic, systemic and histological changes are attenuated at the aforementioned time points, a phenomenon known as the repeated bout effect (RBE; Hyldahl et al., 2017; Nosaka & Clarkson, 1995). The RBE has been suggested to occur due to changes in contractile (Janecki et al., 2014; Prasartwuth et al., 2019), neural (Goodall et al., 2017) and inflammatory (Deyhle et al., 2016) properties along with extracellular matrix remodelling (Hyldahl et al., 2015), with one single factor not being able to explain how recovery is modified (Hyldahl et al., 2017).

Following the performance of a single bout of EE, Dartnall et al. (2009) reported a 40% reduction in motor unit (MU) recruitment threshold in the biceps brachii. Additionally, these authors reported an increase (11%) in MU discharge rate when tested at the same relative force immediately post‐EE (42% decline in maximal voluntary isometric contraction; MVIC), suggestive of a modulation in MU behaviour, possibly to compensate for the loss in force. Furthermore, an increased surface electromyography (sEMG) amplitude *during* EE has been attributed to changes in MU recruitment thresholds, discharge rates and/or increased synchronisation of actively firing MUs (Plattner et al., 2011). Additionally, when comparing an initial fatiguing eccentric and concentric bout of exercise, Jones et al. (2023) reported increases in discharge rate only after EE. However, research investigating single MU behaviour *throughout* the performance of EE is limited.

The performance of EE has been suggested to involve a distinctive neural strategy employed by the central nervous system in contrast to concentric muscle actions, with similar neural drive during submaximal and maximal EE (Duchateau & Enoka, 2016; Enoka, 1996; Pasquet et al., 2006). For example, the reduced firing, increased recruitment and synchronisation of MUs observed during EE arise due to alterations at both supraspinal and spinal levels, representing a specific neural strategy (Duchateau & Enoka, 2016; McHugh et al., 1999). Whilst sEMG activity is increased in the days after EIMD, activity *during* EE is reduced (Enoka, 1996; Komi & Rusko, 1974), and alterations in neural strategy controlling muscle contractions are likely until recovery is accomplished (Taylor et al., 2016). Nardone et al. (1989) reported the activation of ‘high threshold’ MUs during EE, contrasting with their inactivity during concentric exercise. Similar findings were reported by Howell et al. (1995) who reported comparable recruitment patterns to Nardone et al. (1989) in the human first dorsal interosseus muscle. In addition, further support has been provided by McHugh et al. (2000) who showed a higher median frequency of sEMG *during* EE than concentric activity, and hence suggest selective recruitment of higher threshold MUs. These later recruited MUs (higher‐threshold MUs) have been suggested to be selectively activated during, and consequently impaired in the days following, EE (Balshaw et al., 2017; Linnamo et al., 2003; Nardone et al., 1989). Some of these neural alterations have been purported to explain a potential shift in neural strategy during a subsequent bout of EE (Howatson & van Someren, 2007; Warren et al., 2000). A decreased median frequency has been reported to occur without significant adjustment in elbow flexor sEMG amplitude, which has been interpreted as increased activation of lower threshold MUs and/or increased synchronisation during a second bout of EE (Chen, 2003; Howatson & van Someren, 2007). This might demonstrate preferential damage to later recruited type II muscle fibres (Friden & Lieber, 2001), fibres that have been associated with higher threshold MUs in animal models (Jakobsson et al., 1988). Data confirming such associations are scarce in humans (Bigland‐Ritchie et al., 1998), and it is understood that MUs do not strictly associate to a specific fibre type, but rather coexpress in each fibre type (I and II; Trevino et al., 2019). However, it has been suggested that a greater number of higher threshold MUs are present in type II versus type I fibres (Casolo et al., 2020) and it is those fibres that are most susceptible to damage during EE.

Previous work (Dartnall et al., 2008, 2011) has examined changes in MU behaviour following EE using intramuscular EMG. Due to high selectivity, intramuscular EMG only allows the identification of a limited number of MUs during low‐force contractions (Martinez‐Valdes et al., 2016) and it is challenging to track the same MUs across different time points using this method (Martinez‐Valdes et al., 2017). The use of HDsEMG provides an opportunity to detect a greater number of MUs, whilst examining a range of force levels (Holobar et al., 2009). Moreover, it is feasible to longitudinally track MUs within and between sessions (Martinez‐Valdes et al., 2017), affording the capability to assess MU properties during the performance of EE. Given the aforementioned neural characteristics of eccentric muscle contractions and the potent effects of an unaccustomed bout of EE, it is important to study neural responses during the performance of an initial bout of damaging exercise (McHugh, 2003; Pincheira et al., 2020). Previous work has not systematically explored variations in the properties of individual MUs during both an initial and repeated bout of EE. Such an approach could inform the existence of a shift in neural strategies between repeated bouts of EE (Howatson et al., 2007; McHugh, 2003; Pincheira et al., 2018), potentially serving to safeguard the muscle from additional damage (Chen, 2003; Chen et al., 2011). Whilst current research on EIMD has primarily concentrated on changes occurring before and after exercise, limited information exists on the understanding of events during the execution of EE, where damage is induced (Balshaw et al., 2017). Analysing the dynamics of MUs throughout an EE bout will contribute to the understanding of mechanisms underlying EIMD and potential alterations in MU activation strategies (Semmler, 2014). Furthermore, a greater understanding of MU behaviour in response to EE is important to refine interventions for injury prevention, treatment, and resistance training programs. Accordingly, the present study aimed to assess MU behaviour during the performance of an initial and repeated bout of EE.

## 2 METHODS

### 2.1 Participants

Twenty healthy (six females) and recreationally active participants volunteered to take part, all of whom were free from neurological or neuromuscular disorders/muscle injuries and not taking medication that could affect nervous system function. Participant characteristics were (mean ± SD): age 26 ± 5 years, stature 172.7 ± 9.0 cm, and mass 72.3 ± 9.7 kg. Recruitment was determined by a sample size estimation aiming to achieve a statistical power of at least 80%. The purpose was to detect a significant main effect on MVIC impairment following a repeated bout of EE in the dorsiflexors, building upon the observations of Škarabot et al. (2019), who reported MVIC drops of 28% and 20% in Bout 1 and Bout 2, respectively, immediately following EE. In line with principles outlined in the *Declaration of Helsinki*, the study obtained approval from the Northumbria University ethics committee (Reference No. 29440), and all eligible participants provided written informed consent before participating in any aspect of the study. Participants were instructed to avoid engaging in unfamiliar exercises throughout the study duration and to abstain from strenuous activities for at least 1 week before both EE visits.

Inclusion criteria for the study encompassed those who were engaged in recreational physical activity but did not have a background of strength training (i.e., performing resistance exercise <1 time per week). Whilst hormonal contraceptive usage was not an exclusion criterion, an approach was taken to minimise the potential influence of sex hormones on responses to EIMD and central nervous system function (Ansdell et al. 2019; Minahan et al. 2015; Jenz et al., 2023). For premenopausal females taking a monophasic oral contraceptive pill, testing was scheduled on the days of pill consumption. Naturally menstruating females that had a cycle duration range of 26–34 days, and had refrained from contraceptive use for the preceding 6 months were assessed during the early follicular phase (days 1–7) of their menstrual cycles (Ansdell et al., 2020). Lastly, females utilising the contraceptive implant were included, given that this product prevents endogenous hormone concentrations from rising throughout its usage (Hohmann, 2009).

### 2.2 Experimental design

Participants attended the laboratory on three separate occasions, each with a specific purpose. The visits consisted of (1) a familiarisation prior to the damaging protocol, (2) responses measured before, during and after a bout of damaging exercise, and (3) repetition of the second visit after a span of 3 weeks. During the familiarisation session, an introduction to the outcome measures was provided along with a passive demonstration of eccentric activity, to avoid eliciting EIMD. Close attention was given to ensuring that each participant performed the trapezoidal contractions with high accuracy during the familiarisation session. The right dorsiflexors were selected for consistency, as Škarabot et al. (2019) showed no difference in torque measurements for the tibialis anterior (TA) muscle across various time points when considering limb dominance.

### 2.3 Eccentric exercise

During the experimental sessions (Visits 2 and 3), participants completed a series of maximal lengthening contractions on an isokinetic dynamometer (Biodex System 4 Pro, Shirley, NY, USA). Participants sat with their hip and knee angles set at 60° and 110° of flexion, respectively. The foot was placed in a metal plate, fastened to the dynamometer lever arm, with the talus and phalanges secured using non‐compliant Velcro straps. The participants were instructed to focus on activating the TA muscle, concentrating on dorsiflexion. Specifically, they performed 10 sets of 10 maximal lengthening contractions spanning a range of 50° (initiating from 10° of dorsiflexion up to 40° of plantar flexion) at an eccentric angular velocity of 15° s^−1^. During the passive shortening phase, participants were instructed to relax (Power et al., 2012; Škarabot et al., 2019). Peak torque (N m) and total work (J) were recorded across all sets of the eccentric exercise.

### 2.4 Isometric peak torque

The MVIC was derived from the average of three isometric contractions, each lasting 4 s. These contractions were conducted at an ankle joint angle of 30° of plantar flexion (0° = anatomical zero; Power et al., 2012). Analog signals for force and position data were obtained from the dynamometer, digitised (Micro 1401, CED, Cambridge, UK) and synchronised using automated software (Spike2, v8, CED).

### 2.5 Submaximal contractions

The MVIC was measured at baseline and immediately after the bouts of damaging exercise using the calibrated dynamometer whilst MU behaviour was assessed at baseline, halfway through, and after each bout of EE. Prior to measuring peak torque, participants performed a warm‐up consisting of two sets of isometric contractions at perceived intensities of 25, 50, and 75% of maximum. Various ramp contractions were then performed to record HDsEMG signals. Two separate ramp contractions were each conducted twice in a randomised order (20 and 40% of MVIC). Participants gradually increased torque for 5 s, maintained the contraction for 5 s, and then reduced it at the same rate (Del Vecchio et al., 2020).

### 2.6 HDsEMG

After preparing the skin (shaving and cleaning with 70% ethanol), optimal skin–electrode contact was ensured by applying a disposable bi‐adhesive foam layer and filling the electrode holes with conductive paste (AC Cream, OT Bioelecttronica, Turin, Italy). The single adhesive grid consisting of 64 equally spaced electrodes (arranged in 13 rows × 5 columns, 1 mm diameter, 8 mm inter‐electrode distance) was placed over the TA in parallel to the presumed direction of the muscle fibres (Bingham et al., 2017) at approximately 34% between the tibial tuberosity and the intermalleolar line from the tibial tuberosity, over the TA innervation zone (Barbero et al., 2012; Beretta Piccoli et al., 2014). A 64‐channel HDsEMG electrode (OT Bioelettronica) was positioned over the TA with a ground electrode, secured using a moistened strap, attached over the right ulnar styloid process, and a reference electrode was situated over the right medial malleolus (Del Vecchio et al., 2021). The HDsEMG signals were captured in a monopolar configuration, amplified by a factor of ×150, and sampled at a frequency of 2048 Hz (Quattrocento, OT Bioelecttronica). These signals were also subjected to a bandpass filter (10–500 Hz) and acquired using commercially available software (OT, Bioelecttronica). Subsequently, analyses were performed offline using MATLAB (The MathWorks Inc., Natick, MA, USA).

### 2.7 Data analysis

#### 2.7.1 MU analysis

Decomposition of the HDsEMG signals into motor spike trains was accomplished using the convolution kernel compensation method (Holobar & Zazula, 2007). This method's reliability in accurately identifying MU discharge rates, even at high levels of muscle contraction, has been previously demonstrated (Holobar et al., 2014). Following the acquisition of HDsEMG signals, data were analysed offline. This began with decomposition, a process that estimates MU filters, yielding an estimation of a MU spike train (Frančič & Holobar, 2021); bespoke software and coding were utilised (DEMUSE; v5.01, University of Maribor, Slovenia and MATLAB). Decomposed MU filters were then optimised using standardised procedures, segmenting the MU firings from noise/crosstalk from other MUs (Del Vecchio et al., 2020) MUs with a pulse‐to‐noise ratio below 30 dB were excluded and considered unreliable (Holobar et al., 2014). Subsequently, MUs were tracked across time points for each bout by the concatenation of HDsEMG signals, estimation of MU filters from signals recorded during one contraction and their application to the signals recorded during other contractions (Frančič & Holobar, 2021). Duplicates of MUs (30% of the same discharges; tolerance of 0.5 ms) were removed from each visit.

During the ramp contractions, discharge rate at recruitment/derecruitment was calculated as the average of the reciprocal of the first four interspike intervals, and discharge rate at plateau was computed as the average discharge rate at the plateau region of ramp contractions. The coefficient of variation of inter‐spike intervals was calculated at the plateau region of ramp contractions. The recruitment threshold was defined as the torque level at which an MU initiated firing during the ascending phase of the contraction. Conversely, the derecruitment threshold was established as the torque level corresponding to the last firing of a MU during the descending phase of the ramp contraction (Oya et al., 2009).

### 2.8 Statistical analysis

Data for MVIC and total work are presented as means ± SD or 95% confidence intervals (CI) and were analysed using Prism 6 (GraphPad Software Inc., San Diego, CA, USA). Test–retest reliability of pre‐EE MVIC, total work during EE and the number of MUs identified were assessed by determining the absolute and relative typical error (TE). MU characteristics are presented as estimated marginal means (emmeans) and 95% CI and were analysed in R Studio (Posit Software, Boston, MA, USA). For assessment of loss of maximal force and the amount of total work performed between bouts, data were assessed for meeting parametric assumptions; to evaluate normality and variance, the Shapiro–Wilk test and Levene's test were employed, respectively. In case of any violations, appropriate corrections were applied (e.g., Greenhouse–Geisser correction). A split plot in time two‐way repeated measures analysis of variance analysis was then performed. When appropriate, univariate and post‐hoc *t* tests using Bonferroni correction for pairwise comparisons were employed.

Given the diverse nature of the data collected, including distinct individual data points (i.e., MUs) across individuals and groups, utilising mean scores to analyse datasets could obscure the variability in the number of data points stemming from factors like the sampling procedure (i.e., decomposition yield) or physiological influences. Such an approach might inadvertently lead to misinterpretations or concealment of effects (Giboin et al., 2020). Moreover, careful consideration must be given to the interdependence of observations; namely, the behaviour of MUs within an individual can be correlated (Wilkinson et al., 2022). To address these complexities when examining alterations in MU characteristics, such as discharge rate across individuals, a linear mixed model (*lmerTest* package) was used. The model included contraction intensity (two levels: 20 and 40% MVIC), time (three levels: pre‐exercise, during exercise, post‐exercise), bout (two levels: 1 and 2) and their interaction as fixed effects with the participants being accounted for as random intercept. The modelling was conducted separately for each outcome variable (recruitment and derecruitment thresholds, MU discharge rates at recruitment, plateau and derecruitment) with recruitment threshold as a covariate for discharge rate and collectively or individually for each absolute force intensity (20 and 40% MVIC). The statistical significance of the model was evaluated by comparing the fit with and without predictor values through an analysis of variance. Significant main effects were further examined by post‐hoc analyses of estimated marginal means and their 95% CI *(emmeans* package). The level of statistical significance was set at 0.05.

## 3 RESULTS

### 3.1 MVIC and total work

**FIGURE 1:**
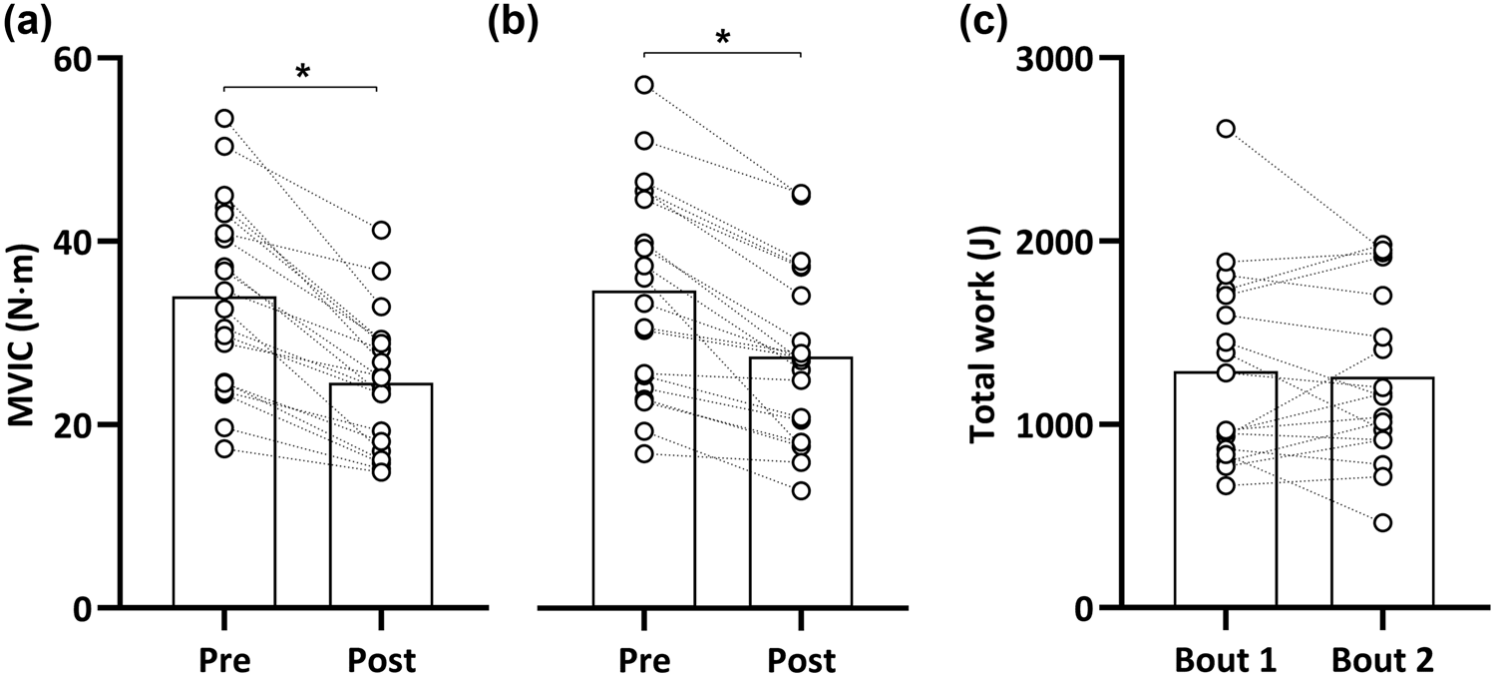
Maximal voluntary isometric contraction (MVIC) pre‐ to post‐damaging exercise in Bout 1 (a) and Bout 2 (b), along with total work (c). Data are presented as bars showing the mean, and individual responses are connected with dashed lines. *P < 0.05, pre versus immediately post.

Maximal voluntary isometric torque and total work were reliable between bouts pre‐EE (MVIC and total work: TE = 2.8 N m, 8% and 197 J, 15%, respectively). Maximal voluntary isometric torque was reduced immediately following the damaging exercise in Bout 1 (34 ± 10 vs. 25 ± 7 N m; *P* < 0.001) and Bout 2 (35 ± 11 vs. 27 ± 9 N m; *P* < 0.001). The pre to post loss in torque was similar in both bouts (Δ9 ± 5 vs. 7 ± 4 N m; *P* = 0.144), as too was the total work produced (1290 ± 521 vs. 1262 ± 470 J; *P *= 0.905; Figure 1).

### 3.2 MU decomposition

The number of MUs identified between bouts pre‐EE was less reliable but remained consistent between bouts (MU number: TE = 4, 18%). Collectively, we identified 1,754 units that were used for analyses across the two contraction intensities, three time points, and two bouts. Four individuals (three females) were removed from analyses due to issues with signal acquisition or inability to identify any MUs; therefore, all data presented hereafter are for 16 participants.

### 3.3 MU activity

Results are derived from tracked MUs at each time point (see Table 1; see Supporting information for results of the accumulation of all recorded MUs that were not tracked). Briefly, the non‐tracked MU data reported within the supplementary material demonstrate similar results to the tracked data explained below.

**TABLE 1:** The population of identified MUs used for analyses with mean (SD) MU yield per participant.

Bout	Contraction	Pre	During	Post	Total	Tracked MUs per subject
Bout 1	20% MVIC	202 (11 ± 5)	100 (6 ± 4)	137 (8 ± 5)	439	6 ± 3
	40% MVIC	202 (12 ± 5)	121 (7 ± 3)	154 (9 ± 4)	477	6 ± 3
Bout 2	20% MVIC	169 (10 ± 6)	98 (6 ± 4)	144 (9 ± 5)	411	6 ± 4
	40% MVIC	171 (10 ± 6)	109 (6 ± 3)	147 (9 ± 4)	427	8 ± 3

Abbreviations: MU, motor unit; MVIC, maximal voluntary isometric contraction.

### 3.4 Coefficient of variation of inter‐spike intervals at plateau

**FIGURE 2:**
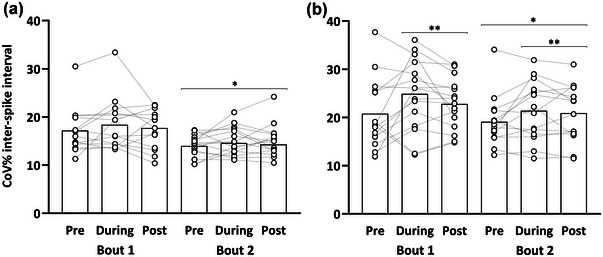
Coefficient of variation (CoV) for inter‐spike intervals during 20% MVIC (a) and 40% MVIC (b) across time points and bouts. Dashed lines represent participant mean responses, whereas the bar plots represent mean group values for each time point. *P < 0.05, Bout 1 greater than Bout 2 at the respective time point. **P < 0.05, greater at the respective time point compared with baseline. MVIC, maximal voluntary isometric contraction.

For coefficient of variation of the inter‐spike interval (CoVISI), there was a main effect of time (*F* = 61.91, *P* < 0.001), bout (*F* = 15.47, *P *< 0.001), and contraction level (*F* = 108.17, *P* < 0.001). This was accompanied by a time × bout interaction (*F* = 3.62, *P = *0.027) and a time × contraction level interaction (*F* = 3.11, *P* = 0.045). A time × bout × contraction interaction was not observed (*F* = 2.81, *P* = 0.061). Post‐hoc tests indicated no differences between time points for the 20% MVIC condition. However, CoVISI was increased for the 40% MVIC condition from pre (19.4 [16.9–22.0]%), during (22.0 [19.5–24.6]%; *P *< 0.001) and post (21.5 [19.0–24.1]%; *P *< 0.001) (Figure 2a). Furthermore, CoVISI was reduced in Bout 2 compared to Bout 1 (20.4 [17.9–22.9] vs. 18.0 [15.5–20.5]%; *P* = 0.018) irrespective of contraction level (Figure 2b).

### 3.5 Discharge rate at recruitment

Discharge rate at recruitment displayed a main effect for time (*F* = 28.6, *P* < 0.001) and contraction level (*F* = 4.64, *P* = 0.03), but not bout (*F* = 0.84, *P* = 0.359). Additionally, there was an interaction for time × bout (*F* = 6.84, *P* = 0.0011). Post‐hoc tests indicated that for Bout 1, discharge rate at recruitment increased by ∼14% from pre to post exercise (9.96 [8.97–11.0] vs. 11.37 [10.38–12.4] pulses per second (pps)B; *P* < 0.001). Furthermore, similar behaviour was present in Bout 2, whereby an increase of ∼5% was evident pre to post exercise (10.25 [9.27–11.2] vs. 10.76 [9.77–11.7] pps; *P* = 0.008). Discharge rate was lower following the second compared to the first bout (11.37 [10.38–12.4] vs. 10.76 [9.77–11.7] pps), respectively (*P* = 0.008).

### 3.6 Discharge rate at plateau

**FIGURE 3:**
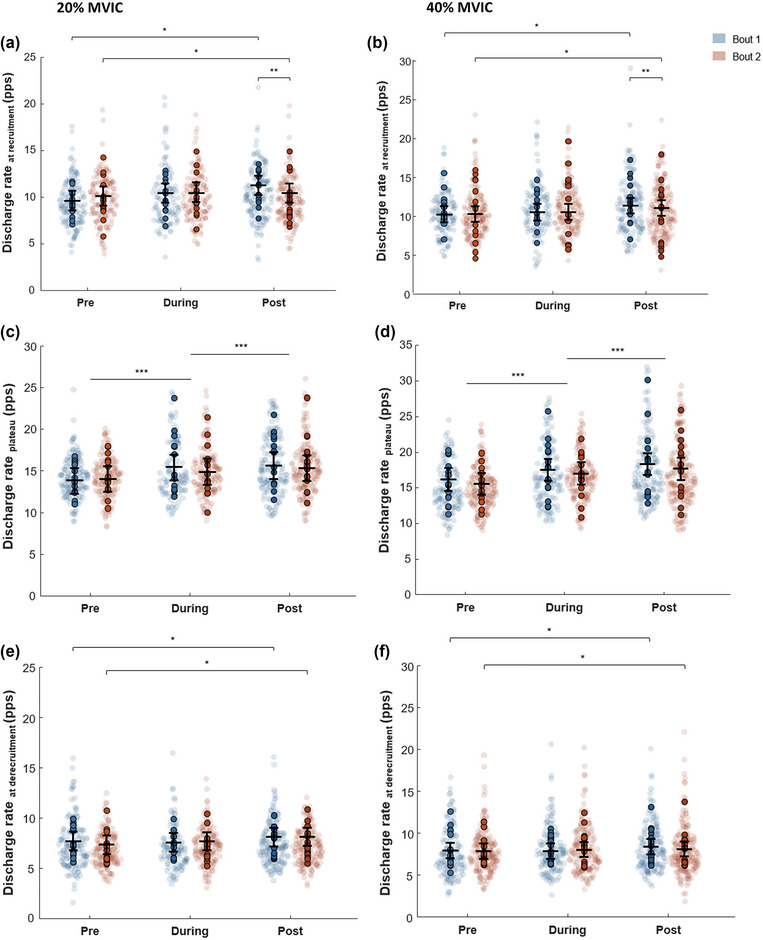
Motor unit discharge rate during recruitment (a, 20% MVIC; b, 40% MVIC), plateau (c, 20% MVIC; d, 40% MVIC) and derecruitment (e, 20% MVIC; f, 40% MVIC). Bout 1 is represented in blue and Bout 2 in red for tracked motor units. The unbounded coloured circles represent individual MU discharge rates for each bout. The bounded coloured circles indicate the individual means for each participant. The horizontal line demonstrates the overall estimated marginal means (emmeans) for each time point, and the whiskers represent associated 95% confidence intervals for each time point. ***P < 0.05, greater at the respective time point compared with baseline; **P < 0.05, Bout 1 greater than Bout 2 at the respective time point; *P < 0.05, between individual time points. MVIC, maximal voluntary isometric contraction.

Discharge rate at plateau displayed a main effect for time (*F* = 134.44, *P* < 0.001), bout (*F* = 15.02, *P* < 0.001) and contraction level (*F* = 375.25, *P* < 0.001). Furthermore, a time point × contraction level interaction (*F* = 3.26, *P* = 0.038) was present. Similar stepwise increases were evident for 20% MVIC and 40% over time (*P* < 0.001, Figure 3). Specifically, during 20% MVIC, discharge rate increased by ∼9% in Bout 1 during (13.9 [12.3–15.4] vs. 15.1 [13.5–16.7] pps) and ∼11% at post (13.9 [12.3–15.4] vs. 15.4 [13.9–17.0] pps, Figure 3c) compared to baseline. For the 40% contraction level, discharge rate at plateau increased by ∼9% from pre‐ to during (15.9 [14.3–17.4] vs. 18.0 [16.5–19.6] pps) and by ∼13% post (15.9 [14.3–17.4] vs. 18.0 [16.5–19.6] pps). These increases were not different between bouts (time × bout × contraction level interaction, *P* = 0.188).

### 3.7 Discharge rate at derecruitment

Discharge rate at derecruitment demonstrated a main effect of time (*F* = 7.48, *P* < 0.001) and contraction level (*F* = 9.85, *P* = 0.002), but not for bout (*F* = 1.42, *P* = 0.233). Post‐hoc analysis revealed that discharge rate increased by ∼8% from baseline to post (7.73 [6.86–8.61] vs. 8.31 [7.25–9.00] pps; *P* = 0.01). Furthermore, no interaction was present between time × bout and contraction level (*F* = 0.19, *P* = 0.830; Figure 3e,f).

### 3.8 Relative recruitment threshold

**FIGURE 4:**
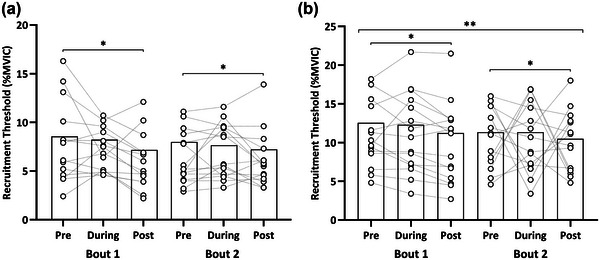
Motor unit recruitment thresholds. Comparison between bouts for relative (% of maximal voluntary isometric force) motor unit recruitment thresholds for both 20% MVIC (a) and 40% MVIC (b). Dashed lines represent participant mean responses, whereas the bar plots represent mean group values for each time point. **P < 0.05, Bout 1 lower than Bout 2 at the respective time point; *P < 0.05, between individual time point. MVIC, maximal voluntary isometric contraction.

Relative recruitment threshold demonstrated main effects for time (*F* = 25.33, *P* < 0.001), bout (*F* = 20.32, *P* < 0.001) and contraction level (*F* = 634.32, *P* < 0.001). Specifically, the recruitment threshold was reduced from baseline to post (10.15 [8.02–12.3] vs. 9.07 [6.94–11.2]%; *P* < 0.001). Furthermore, a bout × contraction level interaction was present (*F* = 4.45, *P* = 0.035). A significant difference at 40% MVIC between bouts was present (12.03 [9.89–14.16] vs. 11.06 [8.92–13.19]%; *P* < 0.001). However, no differences were evident at 20% MVIC (Figure 4a,b).

### 3.9 Relative derecruitment threshold

**FIGURE 5:**
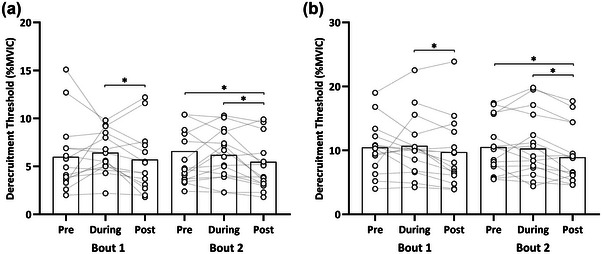
Motor unit derecruitment thresholds. Comparison between bouts for relative (% of maximal voluntary isometric force) motor unit derecruitment thresholds for both 20% MVIC (a) and 40% MVIC (b). Dashed lines represent participant mean responses, and the bar plots represent mean group values for each time point. *P < 0.05, between individual time points. MVIC, maximal voluntary isometric contraction.

For relative derecruitment threshold, a main effect of time (*F* = 25.22, *P* < 0.001), contraction level (F = 852.40, *P* < 0.001) and interaction between time × bout (*F* = 4.07, *P* = 0.017) was present. An ∼10% reduction in relative derecruitment threshold occurred during to post‐exercise in Bout 1 (8.59 [6.81–10.37] vs. 7.73 [5.96–9.51]%; *P* = 0.003). Similarly, a ∼12% reduction was present in Bout 2 at during in respect to baseline (8.24 [6.47–10.02] vs. 7.22 [5.45–8.99]%; *P* < 0.001). Additionally, an ∼16% reduction occurred in Bout 2 post‐exercise in respect to baseline (8.57 [6.80–10.34] vs. 7.22 [5.45–8.99]%; *P* < 0.001) (Figure 5a,b).

## 4 DISCUSSION

The aim of the present study was to assess MU behaviour during an initial and repeated bout of EE. Our results demonstrate that both bouts induced similar decreases in force with no differences in total work performed, implying that the observed differences in MU behaviour were related to an altered neural strategy. Throughout both bouts of EE, stepwise increases in discharge rate accompanied the decline in maximal force. Decreases in relative recruitment threshold immediately after EE was evident in both bouts, but the coefficient of variation of inter‐spike intervals was reduced in the repeated bout. Collectively, this work provides evidence of changes in the neural strategy during the performance of a repeated bout of EE, and provides a plausible explanation of the neural contribution, at least in part, to the repeated bout effect phenomenon.

### 4.1 MU discharge properties

We observed a stepwise increase in MU discharge rate during EE for both bouts, with no differences between bouts. An increase in discharge rate immediately after EE is a well‐established finding (Dartnall et al., 2009; Piitulainen et al., 2012), and our findings are in agreement with Jones et al. (2023) and Hirono et al. (2022) who suggested that an increase in discharge rate of tracked MUs acts as compensation for a reduction in contractile function. Using electrically evoked torque as a measure of peripheral function, these authors reported a reduction in knee‐extensor contractile function post‐fatiguing EE. They suggested that the increased MU discharge rate is caused by increased neural input to maintain force production at any level of contraction (Hirono et al., 2022; Jones et al., 2023). Studies in the dorsiflexors have also suggested that the primary mechanisms for force loss during EE are predominantly peripheral in nature (e.g., decreased twitch torque reduction; excitation–coupling disruption) rather than neural (Baudry et al., 2007; Pasquet et al., 2000). Therefore, increases in discharge rate in the present study likely occurred as compensation for the reduced contractile function (Pincheira et al., 2021), likely evident after each bout as demonstrated by the consistent loss in maximal force (Figure 1).

### 4.2 Coefficient of variation of inter‐spike interval

The CoVISI was increased at the 40% MVIC level during and post both bouts compared to baseline; however, the increase was attenuated in Bout 2. This suggests that as exercise‐induced force reductions manifest during an initial bout of EE, there is increased firing variability and greater synaptic noise. This increased variability has been attributed to alterations in the power of common synaptic input, which causes greater variation in MU input, leading to increased firing variability (Castronovo et al., 2015). Various pathways provide a substantial number of common synaptic inputs to MUs, whereby changes from descending pathways provide excitatory and inhibitory inputs along with peripheral feedback loops (e.g., Ia and III/IV afferents); therefore, various mechanisms of sensory feedback might be responsible for the increased variability (Gandevia, 2001; Garland et al., 1994; Hwang et al., 2020). When a repeated bout was performed, a considerable reduction in CoVISI was present for both contraction levels, suggesting alterations in common synaptic input to the MUs during the performance of a repeated bout of EE. Such alterations are governed by the central descending pathways or decreased sensory feedback via presynaptic inhibition of afferent inputs located at the spinal cord (Gault & Willems, 2013; Taylor et al., 2003). More specifically, our data could be explained by increased excitability of the motor neuronal pool, which is known to gradually increase *after* an initial bout of EE (Duclay et al., 2008; Vangsgaard et al., 2014), causing increased synchronisation between MU firing trains (Negro & Farina, 2012). Kornatz et al. (2005) and Rodriguez‐Falces et al. (2017) found rapid reductions (<2 weeks) in discharge rate variability after lengthening contractions in the first dorsal interosseus muscle. The authors attributed this change to either changes in monoaminergic input from the brainstem or a decreased synaptic noise caused by rapid training adaptations in afferent input. Collectively, our data show that changes in properties of the MU pool are evident when repeated bouts of EE are performed and highlight modifiable neural properties associated with the RBE.

### 4.3 Recruitment threshold and derecruitment threshold

For this study, we normalised the recruitment threshold to a percentage of MVIC to prevent changes in the absolute recruitment threshold from being attributed to decreases in strength after the bouts of EE. A reduction in relative recruitment threshold was present at both contraction levels. This reduction demonstrates that MUs were recruited earlier, immediately after the EE. These findings of a compressed recruitment range after a bout of EE are in agreement with Dartnall et al. (2009) who found a 40% decline in the recruitment threshold of biceps brachii MUs immediately after EE. There was also a non‐significant reduction in the brachialis muscle of 25%, which led to the notion that the behaviour in response to EE could be muscle dependent or due to the variations in the susceptibility to EIMD, which may explain different relative changes. We also observed a decrease (8%) in recruitment threshold pre‐exercise during Bout 2 compared to Bout 1, at the 40% MVIC level, signifying that MUs were recruited earlier in the repeated bout. Consequently, these data imply a shift in recruitment behaviour identified ahead of the repeated bout. A compressed recruitment range indicates a greater number of low‐threshold MUs that contribute to the overall force‐producing capacity (Del Vecchio et al., 2019), and a greater proportion of force output may be reliant on increased firing of low‐threshold MUs (Starbuck & Eston, 2012) – a possible adaptation to protect the muscle from damage (Nardone et al., 1989). Interestingly, significant reductions from baseline in the relative derecruitment threshold were present following the repeated bout, suggesting that MUs were firing longer to maintain force output. This might be attributed to a greater magnitude of persistent inward currents (PICs) in the second bout of EE, which could amplify and prolong synaptic input to generate longer depolarisation of motor neurons (Heckman et al., 2005; Jakobsson et al., 1988; Lee & Heckman, 2000). However, work is needed to confirm the behaviour of PICs following EE.

### 4.4 Limitations and future directions

The TA is a contributor to locomotion, but is not a commonly trained muscle; therefore it is essential to investigate whether these findings apply to larger muscles used in sport and exercise (Byrne et al., 2007), such as the vastii group or bicep brachii. By using a single muscle, the present study has limited the generalisability. The TA was selected due to its substantial MU decomposition yield over other muscles (Del Vecchio et al., 2020). Due to the nature of exercise‐induced muscular fatigue and the reduction in conduction velocity of MU action potential shapes, the identification and reuse of MU filters becomes more complicated. Alterations in muscle geometry and changes in action potential waveforms (Enoka et al., 1992; Frančič & Holobar, 2021) result in a reduced ability to track MUs with fatigue. Therefore, selecting a muscle with the most substantial yield was imperative. It is important to note that a sampling bias could have influenced differential changes in Bout 2 while being tracked at each time point. Nonetheless, the same or similar MUs were likely identified in the second bout and confidence in the data is demonstrated by the similar number of total MUs identified and tracked between bouts.

The notion that there may be divergent alterations in higher and lower threshold MUs during a repeated bout of EE needs to be explored (Macgregor & Hunter, 2018); however, for this study, we decided not to investigate this aspect. Our decision was based upon the need to utilise greater contraction intensities to provide a compelling profile of higher versus lower threshold MUs (Martinez‐Valdes et al., 2020). However, higher contraction intensities (50–70% MVIC), provide complex issues in the accurate quantification of MU spike trains with coincidental fatigue, whereby increased extracellular potassium alters action potential shape and affects pulse‐to‐noise ratios (Del Vecchio & Farina, 2019; Urh & Holobar, 2020). The present sample included both males and females, but given the small sample of females (three with useable data) a sub‐analysis to identify any sex differences was not performed. However, mixed findings regarding sex differences have been postulated after an acute and repeated bout of EE (Bruce et al., 2021; Morawetz et al., 2020; Sewright et al., 2008). Furthermore, given the distinct differences in MU behaviour between sexes (Guo et al., 2022; Jenz et al., 2023; Lulic‐Kuryllo & Inglis, 2022), differences in control strategies may be present to deal with the task requirements of EE.

### 4.5 Conclusions

In summary, we investigated neural changes associated with the performance of repeated bouts of EE. Similar amounts of work performed during the exercise, combined with comparable drops in maximal force post‐exercise, confirm that a consistent exercise stimulus was completed. Some of the MU changes observed during and post‐exercise, such as increased MU discharge rate during the damaging EE in each bout, were likely in compensation for disruption to contractile function and fatigue. A decrease in relative recruitment threshold immediately after EE was present in both bouts compared to baseline, with a reduced recruitment threshold during stronger contractions in Bout 2. Also, reduced derecruitment thresholds were observed immediately after EE in Bout 2 with respect to baseline and the CoVISI intervals was lower in the repeated bout, suggesting reduced firing variability. Collectively, our work provides novel insights into a shift in neural strategy during the performance of a second bout of EE. We observed a greater contribution of lower threshold MUs to force generation among MUs in the second bout, suggesting a likely mechanism contributing to the accelerated recovery known to occur following performance of a repeated bout of EE. Importantly, our data suggest that changes in MU behaviour contribute, in part, to the repeated bout phenomenon immediately upon performing a repeated bout of eccentric activity.

## AUTHOR CONTRIBUTIONS

Oliver Hayman, Paul Ansdell, Luca Angius, Kevin Thomas, Glyn Howatson, & Stuart Goodall conceived the design of the study. Oliver Hayman, Paul Ansdell, Lauren Horsbrough, & Stuart Goodall led or assisted with data collection whilst Oliver Hayman, Paul Ansdell, Luca Angius, Kevin Thomas, Glyn Howatson, Dawson J. Kidgell, Jakob Škarabot, & Stuart Goodall assisted with aspects of data analysis and were involved in revising drafts of the write up, providing intellectual contributions. All authors read and approved the final version of this manuscript and agree to be accountable to all aspects of the work. We ensure that any questions that arise in relation to our work will be appropriately investigated and resolved in a timely manner. All persons designated as authors qualify for authorship, and all those who qualify for authorship are listed.

## CONFLICT OF INTEREST

None declared.

## FUNDING INFORMATION

None.

## Supporting information

Supplementary data for the accumulation of all recorded MUs.

## Data Availability

The data that support the findings of this study are available from the corresponding author upon reasonable request.
