# Supplementary material for: Changes in motor unit behaviour across repeated bouts of eccentric exercise
Source: Exp Physiol. 2024 Sep 3;109(11):1896–908. doi: 10.1113/EP092070 (PMC11522828; doi:10.1113/EP092070)
Supplement: Supplementary file 1 — Supplementary data for the accumulation of all recorded MUs. [file EPH-109-1896-s001.docx]

| **Variable** | **20%** | | | | | |
| --- | --- | --- | --- | --- | --- | --- |
|  | Bout 1 | | | Bout 2 | | |
|  | Pre | During | Post | Pre | During | Post |
| Recruitment threshold (% MVC) | 6.78 (5.11-8.45) | 7.87 (6.17-9.58)* | 6.36 (4.67-8.04) | 5.87 (4.20-7.55) | 7.35 (5.65-9.05) | 6.2 (4.52-7.88) |
| Derecruitment threshold (% MVC) | 5.2 (3.52-6.88) | 6.87 (5.16-8.59)* | 5.94 (4.25-7.64)* | 5.65 (3.96-7.33) | 6.73 (5.02-8.45)* | 5.46 (3.77-7.15) |
| Discharge rate at recruitment (pps) | 9.78 (8.79-10.8) | 10.45 (9.44-11.5)* | 11.36 (10.36-12.4)* | 10.54 (9.55-11.5) | 10.77 (9.76-11.8)* | 10.81 (9.81-11.8)* |
| Discharge rate at plateau (pps) | 13.9 (12.3-15.4) | 15.5 (13.9-17.1)* | 16 (14.4-17.5)* | 14.2 (12.6 – 15.7) | 15.3 (13.7-16.8)* | 15.5 (13.9-17.1)* |
| Discharge rate at derecruitment (pps) | 7.58 (6.88-8.29) | 7.57 (6.84-8.3)* | 8.32 (7.60-9.03)* | 7.43 (6.72-8.14) | 7.86 (7.13-8.59)* | 8.10 (7.39-8.82)* |
| CoV ISI plateau (%) | 17.8 (16.4-19.3) | 19.0 (17.4-20.5) | 18.5 (17.0-20.0) | 15.7 (14.2-17.2) | 16.2 (14.6-17.7) | 16.2 (14.8-17.7) |

**Supplementary Data Table 1.** Properties for non-tracked motor units at each time point for 20 and 40% MVIC.

| **Variable** | **40%** | | | | | |
| --- | --- | --- | --- | --- | --- | --- |
|  | Bout 1 | | | Bout 2 | | |
|  | Pre | During | Post | Pre | During | Post |
| Recruitment threshold (% MVC) | 9.57 (7.91-11.24) | 11.59 (9.90-13.28)* | 9.78 (8.10-11.46) | 9.56 (7.88-11.23) | 10.72 (9.03-12.42)* | 9.67 (7.99-11.35) |
| Derecruitment threshold (% MVC) | 8.64 (6.96-10.32) | 10.86 (9.16-12.56)* | 9.55 (7.86-11.24)* | 9.78 (8.10-11.47) | 10.38 (8.67-12.08)* | 9.16 (7.47-10.85) |
| Discharge rate at recruitment (pps) | 10.46 (9.48-11.4) | 10.98 (9.98-12)* | 11.77 (10.77-12.8)* | 10.79 (9.80-11.8) | 11.08 (10.1-12.1)* | 11.59 (10.6-12.6)* |
| Discharge rate at plateau (pps) | 16.5 (15.0-18.1) | 18.1 (16.6-19.7)* | 18.5 (17.0-20.1)* | 16.4 (14.8-18.0) | 17.6 (16.0-19.2)* | 18.0 (16.5-19.6)* |
| Discharge rate at derecruitment (pps) | 7.64 (6.93-8.34) | 8.09 (7.37-8.81)* | 8.57 (7.86-9.28)* | 7.78 (7.07-8.49) | 7.97 (7.25-8.69)* | 8.28 (7.57-8.99)* |
| CoV ISI plateau (%) | 18.6 (17.2-20.0) | 21.6 (20.1-23.1)* | 21.7 (20.2-23.2)* | 17.5 (16.0-19.0) | 19.3 (17.8-20.9) ***** | 18.9 (17.5-20.4) ***** |

Data are presented as estimated marginal means (95% confidence intervals)* = p < 0.005 when compared to baseline values for the respective bout.

We identified 1,754 units that were used for analyses across the two contraction intensities, three-time points, and two bouts.

**Discharge rate at recruitment**

Discharge rate at recruitment demonstrated a main effect for time (F = 54.21, P < 0.001) and contraction level (F = 28.66, P < 0.001), but not bout (F = 2.29, P = 0.130). Moreover, a time × bout interaction (F = 11.63, P < 0.001) was present. Post hoc tests indicated that irrespective of contraction level, during bout 1, discharge rate increased by ~11% from pre to during (15.2 [13.6-16.8] vs. 16.8 [15.2-18.4] pps, P < 0.001) and pre to post by ~14% (15.2 [13.6-16.8] vs. 17.3 [15.7-18.8] pps, P < 0.001). Furthermore, a similar observation was present for bout 2, whereby discharge rate increased by ~7% from pre to during (15.3 [13.7-16.8] vs. 16.4 [14.9-18.0] pps, P < 0.001) and by pre to post by ~10% (15.3 [13.7-16.8] vs. 16.8 [15.2-18.3] pps, P < 0.001).

**Discharge rate at plateau**

Both contraction levels (F = 924.22, P < 0.001;) demonstrated a stepwise increase in discharge rate over time (F = 246.75, P < 0.001) and between bouts (F = 11.31, P < 0.001). Furthermore, there was a time × bout interaction (F = 6.11, P = 0.002) but despite this, post *hocs* did not reveal any differences (Table 2).

**Discharge rate at derecruitment**

A main effect for time was present (F = 42.91, P < 0.001) and contraction level (F = 10.55, P = 0.001). However, but not for bout (F = 1.42, P = 0.233). Post hoc tests indicated that discharge rates increased at each point in time with respect to baseline. Whereby discharge rate increased by ~3% from pre to during (7.61 [6.92-8.29] vs. 7.87 [7.18 vs. 8.57]; P = 0.006) and by ~9% from pre to post (7.61 [6.92-8.29] vs. 8.32 [7.63-9.01]; P < 0.001).

**Coefficient of variation of inter-spike intervals at plateau**

The coefficient of variation of the inter-spike interval (CoVISI) demonstrated a main effect of time (F = 25.62, P < 0.001), bout (F = 103.51, P < 0.001), and contraction level (F = 101.61, P < 0.001). There was also an interaction between time x contraction level (F = 7.80, P < 0.001) present. *Post hoc* tests indicated that no differences occurred over time for the 20% MVIC. However, for the 40% MVIC level, increases were present when compared to the baseline for all time points (P < 0.001).

**Relative Derecruitment threshold**

A main effect of time (F = 42.26, P < 0.001) and contraction level (F 898.34, P < 0.001) was present, along with a time x bout interaction (F = 11.65, P < 0.001). From pre to during, the relative derecruitment threshold increased in both bouts (Bout 1, P < 0.001; Bout 2, P = 0.003). The increased relative derecruitment threshold remained elevated at post in bout 1 (P < 0.001). however, returned to baseline values at bout 2 post (P > 0.05). Bout 1 pre values were lower than bout 2 pre (6.92 [5.26-8.58] vs. 7.71 [6.05-9.38] %; P < 0.001).

**Relative Recruitment threshold**

A main effect of time (F = 43.08, P < 0.0001), bout (F = 10.0, P < 0.001) and contraction level (F = 653.86, P < 0.001) were present for the relative recruitment threshold. Post hoc analysis demonstrated relative recruitment threshold increased during with respect to baseline (7.94 [6.30-9.59] vs. 9.39 [7.74-11.03] %; P < 0.001). Post-exercise, the relative recruitment threshold returned to baseline (8.00 [6.36-9.65] %). Furthermore, when comparing bout 1 vs bout 2, bout 2 demonstrated lower relative recruitment threshold respectively (8.66 [7.02-10.30] vs. 8.23 [6.59-9.87] %; P = 0.0016).
